# Emergence of livestock-associated MRSA in the Egyptian Nile Delta that carry the exfoliative toxin gene *etA*: a case for enhanced surveillance

**DOI:** 10.1007/s10096-025-05163-z

**Published:** 2025-07-05

**Authors:** Stefan Monecke, Sindy Burgold-Voigt, Sascha D. Braun, Celia Diezel, Elke Müller, Martin Reinicke, Maged El-Ashker, Mayada Gwida, Elisabeth M. Liebler-Tenorio, Samar Boswihi, Muhabat A. Raji, Shahinda Rezk, Abiola Senok, Ali M. Somily, Edet Udo, Ralf Ehricht

**Affiliations:** 1 Leibniz Institute of Photonic Technology (Leibniz-IPHT), Leibniz Center for Photonics in Infection Research (LPI), Jena, 07745 Germany; 2 InfectoGnostics Research Campus, Jena, 07743 Germany; 3 Department of Internal Medicine and Infectious Diseases, Faculty of Veterinary Medicine, Mansoura University, Mansoura, 35516 Egypt; 4 Department of Hygiene and Zoonoses, Faculty of Veterinary Medicine, Mansoura University, Mansoura, 35516 Egypt; 5 Friedrich-Loeffler-Institute (Federal Research Institute for Animal Health), Institute of Molecular Pathogenesis, Jena, 07743 Germany; 6 Faculty of Medicine, Department of Microbiology, Kuwait University, Safat, 13110 Kuwait; 7 Department of Microbiology and Immunology, College of Medicine, Alfaisal University, Riyadh, 11533 Kingdom of Saudi Arabia; 8 Department of Microbiology, Medical Research Institute, Alexandria University, Alexandria, 21561 Egypt; 9 Institute of Virology, Medical Center, University of Freiburg, Freiburg, 79104 Germany; 10 College of Medicine, Mohammed Bin Rashid University of Medicine and Health Sciences, Dubai, United Arab Emirates; 11 School of Dentistry, Cardiff University, Cardiff, CF14 4XY UK; 12 Department of Pathology, College of Medicine, King Saud University and King Saud University Medical City, Riyadh, 11461 Kingdom of Saudi Arabia; 13 Institute of Physical Chemistry, Friedrich-Schiller University, Jena, 07743 Germany

**Keywords:** *Staphylococcus aureus*, MRSA, Exfoliative Toxin A (*etA*), Staphylococcal prophages

## Abstract

**Background:**

*Staphylococcus aureus* is a common opportunistic pathogen. Methicillin-resistant strains, MRSA, carry SCC*mec* elements that include beta-lactam resistance genes *mecA/mecC*. One globally common lineage, Clonal Complex (CC) 15 failed to evolve MRSA until, in 2016, CC15-MRSA were described from Saudi Arabia that carried a SCC*mec* V element also comprising the fusidic acid resistance gene, *fusC*. Henceforth, this strain has spread across Gulf states and Egypt infecting or colonizing both, humans and livestock.

**Methods:**

DNA-microarray-based typing was performed on 134 MRSA isolates collected from livestock and farmers in the Nile Delta region of Egypt in 2022. Isolates with conspicuous toxin gene carriage were sequenced applying Oxford Nanopore Technology.

**Results:**

Twenty-eight out of 134 isolates were assigned to CC15-MRSA-[V + *fusC*]. Thus, this strain was the second most common MRSA strain, behind CC88-MRSA-[IV + *fusC*]. Twenty out of those 28 isolates harboured the gene *etA*, encoding exfoliative toxin A associated with staphylococcal scalded skin syndrome. Sequencing confirmed the presence of *etA* on a *sufB*-integrating prophage. Based on gene content and on electron microscopic morphology after mitomycin C induction, it was assigned to the genus *Phietavirus*. In addition to SCC*mec*-borne genes *mecA, fusC* and *aacA-aphD* (encoding beta-lactam, fusidic acid and gentamicin/tobramycin resistance), isolates also harboured *aadD, lnu*(A), *tet*(K) (for tobramycin, lincosamide and tetracycline resistance) as well as beta-lactamase and cadmium resistance operons on a plasmid. They showed a conspicuous recombination affecting the *hsdS/M* operon associated with the *set/ssl-*locus (νSaα). This, and the identity of the composite SCCmec-[V + *fusC*] element, suggest descent from the *etA*-negative strain previously observed in Gulf states and Egypt.

**Conclusion:**

We describe a novel variant of a CC15 livestock-associated MRSA strain from Egypt. Because of the presence of *etA*, it might be of increased virulence to humans, especially to new-borns who might also be exposed to contaminated milk. Hence, we urgently recommend surveillance of SSSS/Ritter´s disease in Egypt or in people with relevant travel history.

**Supplementary information:**

The online version contains supplementary material available at 10.1007/s10096-025-05163-z.

## Introduction

*Staphylococcus aureus* (*S. aureus*) is a Gram-positive bacterium that frequently colonises skin and mucous membranes of both, humans and animals. It can also cause a wide variety of skin and soft tissue infections, pneumonia, meningitis, endocarditis, mastitis, osteomyelitis, toxic shock syndrome, scalded skin syndrome and sepsis as well as food intoxications. It harbours numerous virulence-associated genes, many of which reside on mobile genetic elements (MGEs) that include phages/prophages, plasmids, pathogenicity islands and others. During the last decades, *S. aureus* also acquired a wide variety of genes causing resistance to antimicrobial agents. The most relevant ones are *mecA* and *mecC* encoding modified penicillin-binding proteins that confer resistance to virtually all beta-lactam antibiotics (with the exception of recently developed compounds such as ceftobiprole and ceftaroline). The *mec* genes are localised on complex MGEs, called staphylococcal cassette chromosome elements (SCC*me*c).

The species *S. aureus* comprises distinct phylogenetic lineages, so-called clonal complexes (CCs) that can be discerned by genotyping methods such as genome sequencing, multilocus sequence typing (MLST) of core chromosomal markers, or by DNA-microarray analyses [1]. These lineages vary in abundance and geographic distribution. CC15 is one of the most common and globally widespread lineages [2–6]. Interestingly, it failed for decades to acquire SCC*mec* elements so that MRSA from this lineage used to be extremely rare. A few isolates of CC15-MRSA have been detected in a collection of Italian MRSA strains sampled in 1980. This included three SCC*mec* I isolates and an isolate described to carry an SCC*mec* I variant in which recombinase genes *ccrA/B*−1 were replaced by *ccrA/B*−2 [7]. Many years later, another CC15-MRSA strain was found in blood cultures as well as retail meat sold in markets in Riyadh, Kingdom of Saudi Arabia [8] and, apparently, also in Iran [9]. The strain from Saudi Arabia was shown by Illumina sequencing and microarray hybridisation to carry a composite SCC*mec* type V element that also included the fusidic acid resistance gene *fusC*, as many MRSA strains from the Middle East nowadays do [8, 10, 11]. In the following years, this strain spread across Middle Eastern and Northern African countries (MENA region). It was observed not only in other regions of Saudi-Arabia [12] but also in the United Arab Emirates [13], in Kuwait [14] and in Egypt. In Alexandria, Egypt, it was not yet observed in 2015 [15] but it accounted for as much as 20% of clinical MRSA isolates in 2020 [16].

During a study of MRSA carriage in small ruminants in the Egyptian Nile Delta region, the authors found a number of *etA*-positive specimens of this strain. This is to be discussed in detail in the present study, as the gene product of *etA* is a clinically highly relevant toxin and as treatment options are limited due to the presence of multiple resistance genes including *mecA.*

*S. aureus* possesses numerous virulence factors, including toxins, adhesion proteins, and biofilm-forming capabilities, which enable it to colonise vertebrate hosts and occasionally, as an opportunistic pathogen, to cause symptomatic infection. Many virulence factors are localised on mobile genetic elements, so that their presence is variable and that they can be found across unrelated phylogenetic lineages of *S. aureus.* One group of these virulence factors are exfoliative toxins. These are serine proteases that specifically cleaves desmoglein 1, a protein in the *stratum granulosum* of the skin. This results in shearing of the *stratum corneum* from the *stratum granulosum* and thus in a formation of blisters in the skin. A localised infection is called bullous impetigo while a generalised, systemic infection is called Staphylococcal Scalded Skin Syndrome (SSSS), with “Ritter´s disease” or “Morbus Ritter von Rittersheim”, “*Schälblattern*” (German for “peeling pox”), “*Dermatitis exfoliativa neonatorum*”, or “staphylococcal Lyell syndrome”, being obsolete, historical or regional synonyms. Symptoms include fever, general malaise, conjunctivitis, facial exanthema, a positive “Nikolsky's Sign” (when slight rubbing of the skin results in shearing of the outermost layer of the skin [17]), formation of liquid-filled or purulent blisters, and peeling or desquamation of the skin. In children, the condition looks dramatic [18] but in most cases [17], it results in complete recovery, adequate care provided. In adults, the mortality is high, up to 60% [16, 17, 19]. Two related exfoliative toxins might play a causative role in these conditions and they do not differ in disease severity, clinical presentation or prognosis. One gene, *etA*, is phage-borne while the other one, *etB*, is carried on a plasmid and, consequently, some *S. aureus* even might carry both ([17, 20], GenBank CP140703.1, CP140676.1, CP140690.1). A third exfoliative toxin gene (*etD*) was discovered in animal strains [21], and it appears not to be associated with SSSS although it has meanwhile also been observed in human strains of *S. aureus* such as the CC80 community-associated MRSA clone [22]. A related gene, *etE* was found in ruminant strains of CC130 [23] and a distinct variant of it from CC152 was associated with a case of necrotising fasciitis in a human [24].

The *etA* gene is uncommon, being found in a range of 0.5 to 5.5% of isolates from carriers or patients with other conditions than SSSS [3, 25–28]. The *etA* gene was not only found in human isolates, but also in bovine isolates of *S. aureus* that were associated with mastitis or cultured from bulk milk [29].

It is localised on, and transmitted, by phages (*Phieta-* or *Dubowvirus*, see below). Thus, it can be found in a number of different CCs of *S. aureus*. CC9, CC15, and CC121 are the most prominent ones, but also CC5, CC6, CC88, and CC1290 might carry *etA* (for GenBank entries, see Fig. 2 and Supplemental Files 5a/b). It also has been found, very sporadically, in MRSA, belonging to CC5-MRSA-V from Pakistan (GenBank CP039162.1), CC88-MRSA-IV from Kenya (CP141435.1 and CP141460.1), CC121-MRSA-IV from Japan [30], CC121-MRSA-VT from Australia ("WA MRSA-022"; [31] and G. Coombs, Perth, WA, pers. comm.) and CC398-MRSA-IV from China [32]. To the best of our knowledge, this toxin gene has not yet been observed in CC15-MRSA.

In the present study, we investigate two essentially identical genome sequences of a novel livestock-associated CC15-MRSA harbouring *etA*, found in humans and small ruminants from the Egyptian Nile Delta.

## Materials and methods

### Isolates

Microarray-based typing was performed on MRSA isolates collected from livestock and farmers in the Nile Delta region of Egypt in 2022. For the study, informed consent for the investigation of staphylococci was obtained from the owners. All applicable international guidelines for the care and use of animals were followed. Milk samples (10 mL) were collected from all functional udder quarters in sterile Falcon tubes, excluding initial streams. Samples were promptly transported to the laboratory in a cooler, typically within two hours. Each sample was mixed with an equal volume of trypticase soy broth and incubated for 24 h at 37 °C. Subsequently, a loopful (approximately 10 μL) of each sample was streaked onto Baird Parker or Oxacillin-Mannitol agar plates and incubated at 37 °C for 24–48 h. Colonies exhibiting typical staphylococcal growth were transferred to sheep blood agar (Oxoid, Wesel, Germany) and incubated at 37 °C for 24 h. Cultivated colonies were examined for purity and confirmed as *Staphylococcus spp*. through routine bacteriological and biochemical tests. Selected and re-cloned strains were preserved as glycerol stocks at −20 °C for further analysis.

Isolates that were identified as MRSA were sent to the Jena working group for microarray-based characterisation (see below). Twenty-eight out of 134 MRSA isolates from humans, from milk samples of small ruminants and from farm cats, were assigned to CC15-MRSA-[V + *fusC*]. Thus, this strain was the second most common MRSA strain in the collection from this study, behind CC88-MRSA-[IV + *fusC*]. Twenty out of 28 isolates, originating from humans and from milk of mastitic small ruminants, harboured, according to DNA-microarray analysis (see below), the gene *eta*, encoding exfoliative toxin A. This toxin was previously observed in CC15, but not in CC15-MRSA. Hence, two isolates were sequenced applying Nanopore technology (see below).

The MRSA isolate 012ON104 was recovered from a milk sample of a recently parturient Baladi goat exhibiting subclinical mastitis in summer 2022. The goat, weighing 30 kg and aged 4 years, was part of a household flock in Belqas District, Dakahlia Governorate, northeast of Cairo. Despite appearing healthy, the goat experienced reduced milk production, though milk consistency remained normal. Vital signs were within normal ranges. The udder appeared normal but smaller, with diagnosis confirmed by a positive California Mastitis Test and somatic cell count exceeding 200,000 cells mL^−1^.

The MRSA isolate 140ON271 originated from a milk sample of sheep that had an acute clinical mastitis attack. The 3-year-old ewe, weighing 60 kg, belonged to a sheep flock in Gamasa, Dakahlia Governorate, Egypt. The ewe was primiparous and demonstrated cardinal signs of inflammation in one udder’s quarter, in addition to fever, depression, anorexia, and abnormal physical characteristics of milk, such as discoloration, aberrant smell, and alterations in viscosity, texture, and taste.

Both flocks were managed under intensive farming conditions with free-stall housing and manual milking. Regular vaccinations against Peste des petits ruminants, bluetongue, and *Pasteurella spec.* were administered, along with deworming treatments. The flocks followed similar rural smallholder farming practices, with animals primarily fed grasses, cultivated fodder, and crop residues. Local veterinary records indicated previous use of antibacterial agents like penicillin, oxytetracycline, and marbofloxacin for unrelated ailments.

For comparison and in order to investigate possible phylogenetic relations, one of the original Saudi CC15-MRSA-[V + *fusC*] isolates was sequenced as well as a clinical isolate from Kuwait. The Saudi isolate originated from a blood culture, sampled in November 2013 (RUH-2 = NIVU_13; SAMN06925302). It was previously sequenced using Illumina technology, resulting in a fragmentated sequence, and fragmentation into several contigs hindered a description of its SCC element [8, 10, 11]. The Kuwaiti isolate 6133 originated from a diagnostic sample collected in 2020 or 2021 (Kuwait-06133). A second clinical isolate from Kuwait, 25912, was included because it had, according to the microarray analysis, a “PseudoSCC*mec* class C” element, *i.e*., an SCC*mec* like element that lacked *ccrAA/C* recombinase genes, and it was also negative for *fusC*.

### Microarray-based molecular characterisation

A characterisation of the MRSA isolates from Egypt was performed using a DNA-microarray-based assay (INTER-ARRAY, Bad Langensalza, Germany). This included the detection of resistance genes, virulence markers and other genes of interest as well as an assignment to clonal complexes, strains and SCC*mec* types. The microarrays, related protocols and methods as well as probe and primer sequences have previously been described in detail [1, 33, 34]. Briefly, *S. aureus* was re-cloned on Columbia Blood Agar. Colonies were harvested after overnight incubation and DNA extraction was performed with an enzymatic combination of lysis enzymes kit buffer and Qiagen DNA extraction columns (Qiagen, Hilden, Germany. A multiplexed linear amplification for all targets covered was performed using one specific primer per target. During the amplification, biotin-16-dUTP labels were incorporated into the amplicons. After incubation and several washing steps, the hybridisation to the probes on the array was performed and streptavidin horseradish peroxidase was added that bound to the biotin labels incorporated into the amplicons. The peroxidase caused a local precipitation of a dye added as last step at those spots where the specific amplicons were bound to the probes. Microarrays were photographed and analysed with a designated reader and software (INTER-ARRAY).

### Whole-genome sequencing

For sequencing, overnight cultures of *S. aureus* strains where grown on Columbia Blood Agar plates (Becton Dickinson GmbH, Heidelberg, Germany) at 37 °C. To obtain longer reads, whole genome DNA extraction was performed using the NucleoBond High Molecular Weight DNA Kit (Macherey–Nagel, Düren, Germany) following the manufacturer’s instructions. As recommended, enzymatic lysis with lysostaphin was included at the beginning of the protocol.

Whole genome sequencing of *S. aureus* isolates was performed using the MinION platform (Mk1B; Oxford Nanopore Technologies, ONT, Oxford, UK). Library preparation was conducted with the ONT ligation-native barcoding kit (SQK-NBD112.24), including an Agencourt AMPure XP (Beckman Coulter, Brea, CA, USA) purification step at a 1:1 ratio (v/v) prior to library preparation. The initial step of DNA repair and A-overhang generation was carried out with extended incubation times for improved efficiency. All other steps were performed following the ONT library preparation protocol. An initial flow cell check identified approximately 1,600 active pores before sequencing. Library quantification was performed using a Qubit 4 Fluorometer (ThermoFisher Scientific, Waltham, MA, USA), and approximately 100 ng of DNA per strain was loaded onto a FLO-MIN114 flow cell. Sequencing was conducted for 72 h using MinKNOW software (v24.02.16). Basecalling was performed with the Dorado basecaller (v0.5.1; Oxford Nanopore Technologies), converting MinION raw reads (POD5) into quality-tagged sequence reads while applying barcode trimming with the model *dna_r10.4.1_e8.2_400bps_sup.*

Assembly of quality-tagged reads into complete, circular contigs was carried out using Flye software (v2.9.1). The assemblies were then polished in two stages: first, through four iterative rounds of Racon (v1.5.0) with parameters (-m 8—× 6 -g 8 -w 500), followed by Medaka (v1.12.1) using the model *r1041_e82_400bps_sup_v4.3.0.*

### MLST, *spa*-typing and *dru*-typing

MLST sequence types, *spa*- and *dru-*types were deduced from whole genome sequences using freely accessible websites https://pubmlst.org/bigsdb?db=pubmlst_saureus_seqdef&page=sequenceQuery for MLST, https://spatyper.fortinbras.us/ for *spa*-typing and http://drutyping.org/wordpress/ for *dru-*typing (accessed 2025, Jan 30th and Feb 2nd).

### Prophage induction and phage DNA isolation

Phages were induced as previously described [35–37]. For this purpose, bacterial cultures of 012ON104 and140ON271 were grown overnight in 2 × TY medium and subsequently cultivated at 37 °C until they reached the mid-exponential growth phase (t = 2 h, OD = 0.34 and 0.50 for 012ON104 and140ON271, respectively). For induction, mitomycin C (Roche, Basel, Switzerland) was added to a final concentration of 0.5 μg/mL, whereupon cultivation was continued at 30 °C until a decrease in optical density (OD at 600 nm) was evident compared to the previous measurement (t = 5 h, ΔOD = 0.2 and 0.6 for 012ON104 and140ON271, respectively).

The resulting lysate was centrifuged at 4 °C and 3000 × g. The supernatant was neutralised with 0.1 N NaOH and filtered through a 0.20 µm cellulose acetate (CA) membrane filter (Sartorius, Göttingen, Germany). To isolate the phage DNA (p-DNA), the phage filtrate was centrifuged again at 4 °C and 3000 × g for 30 min. Subsequently, the supernatant was treated with 10 µg/mL DNAse I (NEB, Frankfurt/Main, Germany) and 10 µg/mL RNAse (QIAGEN, Hilden, Germany) for one hour at 37 °C. Then, 20 mM EDTA, 50 µg/mL Proteinase K and 0.1% SDS were added stepwise and the mixture was incubated for another hour at 65 °C and 550 rpm. Subsequently, a phenol–chloroform DNA extraction was performed according to a proven method [38]. The final DNA concentration was determined to 6.5 ng/µl and 6.0 ng/µl for for 012ON104 and140ON271, respectively, using a Qubit 4 fluorometer (ThermoFisher Scientific) according to the manufacturer's instructions.

### Sequencing of phage preparations

Following extraction, DNA was prepared for sequencing using the 1D Genomic DNA Ligation Kit (SQK-NBD114.24; Oxford Nanopore Technologies) in accordance with the manufacturer’s protocol. Before library preparation, DNA was purified with an Agencourt AMPure XP (Beckman Coulter) step at a 1:1 (v/v) ratio. Approximately 600 ng of DNA per sample, quantified using a Qubit 4 Fluorometer (Thermo Fisher Scientific), were used for library preparation. DNA repair and A-overhang generation were conducted in a single step using the NEBNext FFPE DNA Repair Mix and the NEBNext Ultra II End Repair/dA-Tailing Module (New England Biolabs, Ipswich, MA, USA), with tripled incubation times for enhanced performance. A second purification step was included to facilitate barcoding before adapter ligation. The library was loaded onto an Oxford Nanopore Technologies flow cell (FLO-MIN114, featuring R10.4.1 pores).

Sequencing was performed on a MinION Mk1B device for 90 h, starting with approximately 1500 active pores and managed via MinKNOW software (v24.02.16).

Basecalling was conducted using the dorado basecaller (v0.5.1) with the model *res_ dna_r10.4.1_e8.2_400bps_sup@v5.0.*0, generating FastQ files with 4000 reads per file. Barcode trimming was carried out with dorado demux (v0.7.3) using the same model.

Contig assembly of quality-filtered sequence reads for each strain was performed with flye software (v2.9.1). The assembled contigs were polished in two stages: first, through four iterative rounds of racon (v1.5.0) with parameters match 8, mismatch 6, gap 8, and window length 500, followed by final polishing with medaka (v1.12.1) using the model *r1041_e82_400bps_sup_v5.0.0.*

### Phage detection by transmission electron microscopy (TEM)

The negative staining procedure of the phage preparations was performed as previously described [39]. In short, 400-mesh copper grids filmed with formvar and coated with carbon were hydrophilised by glow discharge immediately prior to use. The phage preparations were vortexed and 30 µl-drops placed on dental wax plates. Copper grids were incubated on the drops for 30 min. After washing with distilled water, they were put on a drop of 1% uranyl acetate for 1 min for contrasting. Grids were examined in a transmission electron microscope (Tecnai 12, FEI Deutschland GmbH, Dreieich, Germany) and micrographs of at least 30 phage particles were taken with a digital camera (TEMCAM FX416, TVIPS, Gauting, Germany). Particle sizes were measured using the EM-Measure software (TVIPS).

## Results

### MLST and* spa* types

The analysis of the sequences of the two Egyptian *etA*-positive CC15-MRSA-[V + *fus*] yielded a MLST sequence type (ST) 1535 (13–13–206–1–12–11–13), with the closest core genome MLST (cgMLST) profile being cgST-19159 (at 34 and, respectively, 35 mismatches). The *etA*-negative putative ancestor strain, represented by RUH-2, Kuwait-06133 and Kuwait-25912, also belonged to ST1535.

The RIDOM *spa* type of all five isolates was t084, repeat succession: 07–23-1234-34–12-12–23-02–12–23, which is a common *spa* type in CC15 (https://spa.ridom.de/spa-t084.shtml; accessed at 2025, Jan 30th).

### Description of the SCC*mec* element

**Fig. 1:**
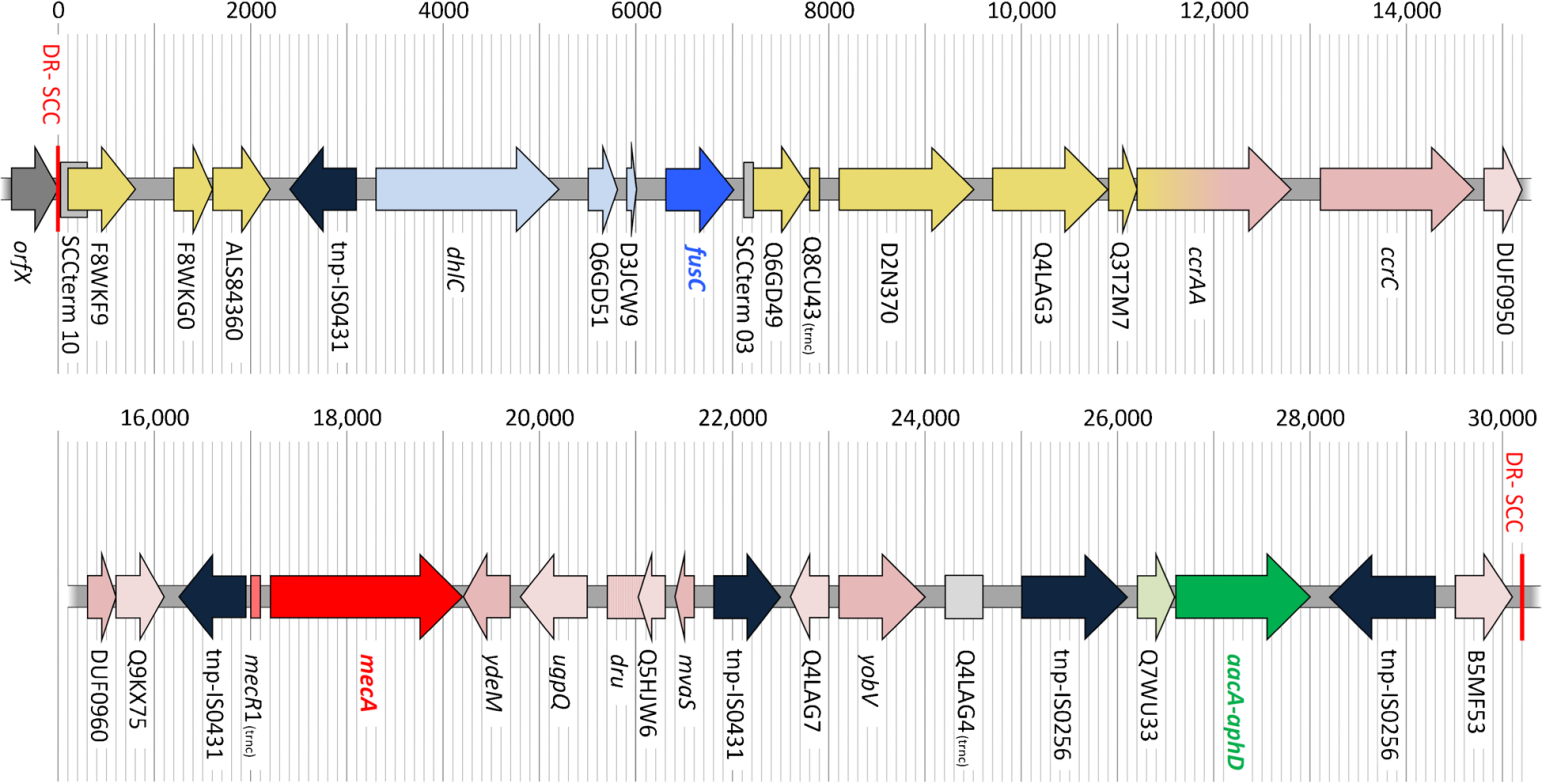
The SCC*mec* element in the Egyptian isolate 012ON104, 140ON271, RUH-2 and Kuwait 06133. The *mecA* gene is red, the associated genes pink; *fusC* and associated genes are shown blue. The aminoglycoside resistance gene *aacA-aphD* is shown in green and transposase genes in dark blue. The genes known from GenBank AP019306.1 and CP128651.1 (see text) are displayed in yellow. The SCC*mec* sequence of Kuwait-25912 lacked all genes between the two reversely orientated *tnp-*IS0431 copies and had also only a single copy of that gene.

The SCC*mec* element of the two Egyptian isolates, of RUH-2 and of one of the two Kuwaiti isolates (Kuwait 06133) is a composite element comprising of elements of diverse backgrounds. Gene content and exact locations of genes detected are provided in Table 1; a schematic diagram is shown in Fig. 1.

**Table 1:** The SCC*mec* element in CC15-MRSA-[V + *fus*]

Gene ID	Gene product/explanation	Position in 012ON104	Orientation	140 ON271	RUH-2	KWT-06133	KWT-25912	*S. haemolyticus* F0718, CP128651.1	SCC*mec/fus* from RUH-32, MK991791	CC1-MRSA, CP113244.1
*orfX*	23S rRNA pseudouridine -methyltransferase RlmH	(35,614..36,093)	forward	X	X	X	X	(2,358,559..2,359,038)	(1..480)	(33,687..34166)
SCCterm10	Terminus of SCC towards *orfX*	(36,094..36,394)	N/A	X	X	X	X	(2,358,258..2,358,558)		
F8WKF9	Putative membrane protein	(36,159..36,935)	forward	X	X	X	X	(2,357,717..2,358,493)		
F8WKG0	Putative exported protein	(37,307..37,690)	forward	X	X	X	X	(2,356,962..2,357,345)		
ALS84360	Unknown SCC*mec*-ass. marker, AUC50 RS00145	(37,702..38,334)	forward	X	X	X	X	(2,356,318..2,356,950)		
tnp-IS0431	Transposase for IS6,IS431,IS257; MW0027,SAR0687	(38,497..39,171)	reverse	X	X	X	X	(2,355,482..2,356,156)		
*dhlC*	ATP-dependent DNA helicase	(39,349..41,289)	forward	X	X	X	-		(19,244..21,184)	
Q6GD51	Putative protein	(41,546..41,854)	forward	X	X	X	-		(21,441..21,749)	
D3QFP0-SCC	Putative lipase/protease (not shown in Fig. 1 because of overlap with D3 JCW9)	(41,901..42,139)	reverse, truncated	X	X	X	-		(21,796..22,034)	Present but in different localisation
D3JCW9	Putative protein	(41,964..42,122)	forward	X	X	X	-		(21,859..22,017)	Present but in different localisation
*fusC*	Fusidic acid resistance protein C	(42,440..43,078)	forward	X	X	X	-		(22,335..22,973)	Present but in different localisation
SCCterm03	Terminus of SCC towards *orfX*	(43,171..43,257)	N/A	X	X	X	-	(2,346,016..2,346,102)	(23,571..23,658)	Present but in different localisation
Q6GD49	Putative protein within SCC	(43,258..43,887)	forward	X	X	X	-	(2,345,386..2,346,015)	(23,659..24,288), different allele	
Q8CU43	Putative protein within SCC	(43,905..44,022)	forward, truncated	X	X	X	-	(2,345,251..2,345,368)	(24,303..24,545), different allele	
D2N370	Putative protein	(44,153..45,628)	forward	X	X	X	-	(2,343,645..2,345,120)		
Q4LAG3	Bifunctional exonuclease/DNA polymerase	(45,855..46,955)	forward	X	X	X	-	(2,342,318..2,343,418)		(37,511..38,611), different allele
Q3T2M7	Putative protein	(46,948..47,316)	forward	X	X	X	-	(2,341,957..2,342,325)		(38,604..38,972), different allele
*ccrAA*	Cassette chromosome recombinase homologue	(47,316..48,935)	forward	X	X	X	-	(2,340,314..2,341,957), see text and Suppl. 2		(38,972..40,591), see text and Suppl. 2
*ccrC*	Cassette chromosome recombinase C	(49,161..50,837)	forward	X	X	X	-	(2,338,410..2,340,089), different allele		(40,817..42,493)
DUF0950 = Q93IE0	Protein of uncharacterised function/uracil-DNA glycosylase inhibitor	(50,943..51,281)	forward	X	X	X	-			(42,599..42,937), different allele
DUF0960 = Q0P7G0	Putative protein/pyridoxal phosphate-dependent enzyme	(51,377..51,688)	forward	X	X	X	-			(43,033..43,344)
Q9KX75	Putative proteinDUF1643 domain-containing protein	(51,704..52,210)	forward	X	X	X	-			(43,360..43,866)
tnp-IS0431	Transposase for IS6,IS431,IS257; MW0027,SAR0687	(52,359..53,033)	reverse	X	X	X	-			(44,015..44,689)
*mecR*1_trunc	Methicillin resistance repressor 1, truncated variant associated with SCC*mec* V	(53,074..53,181)	reverse, truncated	X	X	X	X			(44,730..44,837)
*mecA*	Alternate penicillin-binding protein 2, methicillin/beta-lactam resistance	(53,281..55,287)	forward	X	X	X	X			(44,937..46,944)
*ydeM*	Putative dehydratase	(55,333..55,761)	reverse	X	X	X	X			(46,990..47,418)
*ugpQ*	Glycerophosphoryl diester phosphodiesterase	(55,858..56,601)	reverse	X	X	X	X			(47,515..48,258)
*dru*−09	SCC direct repeat units, ninefold (see text)	(56,803..57,280)	N/A	X	X	X	X			(48,460..48,737), dru-06, see text
Q5HJW6	Putative membrane protein	(57,110..57,420)	reverse	X	X	X	X			(48,607..48,877)
*mvaS*-SCC	Truncated 3-hydroxy-3-methylglutaryl CoA ligase-synthetase	(57,518..57,907)	reverse	X	X	X	X			(48,975..49,364)
tnp-IS0431	Transposase for IS6,IS431,IS257; MW0027,SAR0687	(57,943..58,617)	forward	X	X	X	X			(49,400..50,074)
Q4LAG7	Putative protein (SCC*mec*V/VT-specific allele)	(58,677..59,105)	reverse	X	X	X	X			(37,511..38,611)
*yobV*	Regulatory protein (SCC*mec*V/VT-specific allele)	(59,186..60,115)	forward	X	X	X	X			Replaced by tnp-IS0256 and an integrated plasmid
Q4LAG4	Putative protein associated with SCC*mec*V/VT	(60,277..60,741)	forward, truncated	X	X	X	X			
tnp-IS0256	Transposase for insertion sequence IS256, SAT0131_00065	(61,061..62,232)	forward	X	X	X	X			
Q7WU33	GNAT family acetyltransferase	(62,277..62,681)	forward	X	X	X	X			
*aacA-aphD*	Aminoglycoside phosphotransferase, gentamicin/tobramycin resistance	(62,682..64,121)	forward	X	X	X	X			Present but in different localisation
tnp-IS0256	Transposase for insertion sequence IS256, SAT0131_00065	(64,251..65,423)	reverse	X	X	X	X			Present but in different localisation
B5MF53	Putative protein	(65,605..66,177)	forward	X	X	X	X			
DR_SCC	Direct repeat of SCC	(66,240..66,258)	N/A	X	X	X	X			

The positions and orientations columns refer to the genome sequence of 012ON104. The following four columns show presence (“X”) or absence (“- “) in the other study strains. The last three columns show homologous and contiguous regions from other sequences. For a diagram of the SCC element in CC1-MRSA, CP113244.1, see [16]

The first part immediately downstream of the *orfX* gene comprises a series of genes [F8 WKF9 + F8 WKG0 + ALS84360] followed by a transposase gene, *tnp*-IS0431. This group of genes, including the terminus of SCC towards *orfX* (“SCCterm 10”; AB505630.1:[581..881]) can also be seen in some SCC*mec* elements of *S. aureus* (*e.g*., the CC398 strain RIVM3897, GenBank CP013621.1, the Japanese CC5-MRSA strain TUM9463, GenBank AP019306.1, that also includes SCC*mec* II and ACME) as well as in the *Staphylococcus haemolyticus* strain F0718 (GenBank CP128651.1, that has an SCC element without *mecA/C* genes but comprising CRISPR/*cas* and *kdp*-SCC operons). Usually, it is followed by heavy metal resistance genes which, however, are absent here.

The region comprising of [*dhlC* + Q6GD51 + D3JCW9 + *fusC* + SCCterm3 + Q6GD49 + Q8CU43] can be found in several SCC*mec*/SCC*fus* composite elements [11], namely in the CC30 strain RUH-32, GenBank MK991791.1 [40] where it is combined with something resembling an SCC*mec* VI element. MK991791.1, however, has a ca. 500 bp insert between *fusC* and the terminal SCC sequence fragment (“SCCterm 3”; FR753166.1:[481..568]) which was absent from all CC15 sequences. A shorter variant, [*dhlC* + Q6GD51 + D3JCW9 + *fusC*] is present in the composite SCC element of the CC779 strain M06/0171 (GenBank HE980450.1; [41]) as well as in \ lineages [40]. The gene downstream of *fusC*, Q6GD49, might originate either from a F0718-like SCC element (*e.g.,* CP128651:[2,345,386..2,346,015]) or from a SCC*fus* (BX571857:[54,144..54,773]). The allele in the CC15-MRSA is completely identical to the former, but differs in 30 out of 630 nucleotides from the latter. Similarly, the following ca. 120 bp (that might constitute a truncated variant of Q8CU43) match the F0718-allele better than the one associated with SCC*fus*. The next gene, encoding a putative protein/DUF6038 family protein, D2 N370, again, is also present in F0718, (CP128651:[2,343,644..2,345,120]) but absent from SCC*fus* elements. In conclusion, it can safely be assumed that a SCC*fus* element such as the one from RUH-32 or M06/0171 integrated into a pre-existing F0718-like SCC element replacing or removing several of its genes (CP128651: QU662_11585 to QU662_11630).

The following genes, coding for “putative proteins” Q4LAG3 and Q3T2M7, as well as the recombinase homologue *ccrAA* could originate from either, a F0718-like SCC region or from SCC*mec* V such as the one from an Egyptian CC1-MRSA strain (Alexandria 2020–19, GenBank CP113244.1, [16], see below). The alleles of the former two genes indicate the first possibility. In *ccrAA* gene of CC15-MRSA-[V + *fusC*], the 5´-end (*ca.* 550 of 1620 bp) differ in a series of mismatches from the one of CP113244.1 but resembles more closely the corresponding part of the gene from *S. haemolyticus* F0718, GenBank CP128651.1. Approximately from position 550 to position 1180, sequences from CC15-MRSA study isolates, F0718, and CP113244.1 are all virtually identical, while from position 1180 to end, the study isolate sequences clearly match CP113244.1 (for an alignment, see Supplemental File 1) while other *ccrAA* sequences differ over their entire lengths. This suggests a recombination event involving CP128651- and CP113244-like progenitor sequences with recombination breakpoint or “fault line” bisecting *ccrAA*.

The following genes including *ccrC*, truncated *mecR1*, *mecA*, *ydeM*, *ugpQ*, Q5HJW6, truncated *mvaS*-SCC, tnp-IS0431 (= MW0027), Q4LAG7, and *yobV* all can, completely or partially be found in MRSA with complex, composite SCC*mec* elements. Most genes are also present in SCC*mec* VT elements, but the order and orientation of genes is usually different. The abovementioned CC1-MRSA from Egypt (CP113244.1), however, harbours the same genes in the same order (see Table 1) and relative orientation. This strain also harbours *fusC* and *aacA-aphD* albeit at other positions, in a different context. The *dru* region of all CC15-MRSA-[V + *fusC*] sequenced herein spanned 478 bp, corresponding to eleven repeat units with a *dru* repeat pattern [5a-2d-3i-0−2d-5b-3a-2g-6o-4e-3e] corresponding *dru* type dt11dc. CP113244.1 has a shorter *dru*, in which the some of the repeats are identical 5a-2d-0-6o-4e-3e]. The two genes (Q4LAG7 and *yobV*, a"transcriptional regulator"gene) at the downstream end of this region could either be associated with *fusC* or with SCC*mec* elements. The alleles of both genes observed in the study strains indicate that they belonged to the latter. Finally, an integrated transposon followed that carries the gentamicin/tobramycin resistance gene *aacA-aphD*.

### The recombinant* hsdM/S-ssl *(νSaα) locus

As previously described, the *hsdM* gene and the 5'-part of the *hsdS* gene of *set/ssl*-locus (νSa*α*) of the original Saudi strains were replaced by their paralogs from the *spl* locus (νSaβ). This was confirmed by Nanopore re-sequencing of RUH-2. The Egyptian and Kuwaiti isolates described herein share this chimeric form of the *hsdS-ssl* gene, and they also have a *hsdM-spl/*νSaβ*-*like gene in position (νSaα) of the *hsdM-ssl* gene.

While for *hsdM*, the difference lies only in several single nucleotide polymorphisms (Supplemental File 2), the recombination was most conspicuously visible due to the different length of the *hsdS-ssl* gene in these CC15-MRSA (1185 nt), while CC15-MSSA normally have a *hsdS-ssl* gene of 1170 nt (GenBank CP120001, CP120009, CP120023, CP012970, CP012972, CP151815, LT963437) while all CC15-MRSA strains examined have a *hsdS-ssl* (νSaβ) gene of 1236 nt (Supplemental File 3). Two previously published CC15-MSSA sequences (CP149458.1, CP152328.1), however, share *hsdM/S-ssl* alleles with the CC15-MRSA sequences.

### Plasmids

The two Egyptian strains carried the same plasmid which was in both cases represented by one circular contig of 35,258 bp length (in isolate 12ON104 or, respectively, 35,253 bp in isolate 140ON271). It comprised the penicillinase operon *blaZ/R/I*, the aminoglycoside resistance gene *aadD*, the lincosamide nucleotidyltransferase gene *lnu*(A) (formerly known as *linA*), the tetracycline efflux/transporter gene *tet*(K) as well as the heavy metal/cadmium resistance operon genes *cadD* and *cadX*. The other three isolates had similar plasmids. Most notably, in RUH-2, *tet*(K), was absent and in Kuwait-25912, a part of the plasmid was inverted. In Kuwait-6133, the plasmid sequence measured as much as approximately 69,000 bp with the entire set of genes being duplicated, but this might have been an artefact.

### Analysis of the prophage sequences

**Fig. 2:**
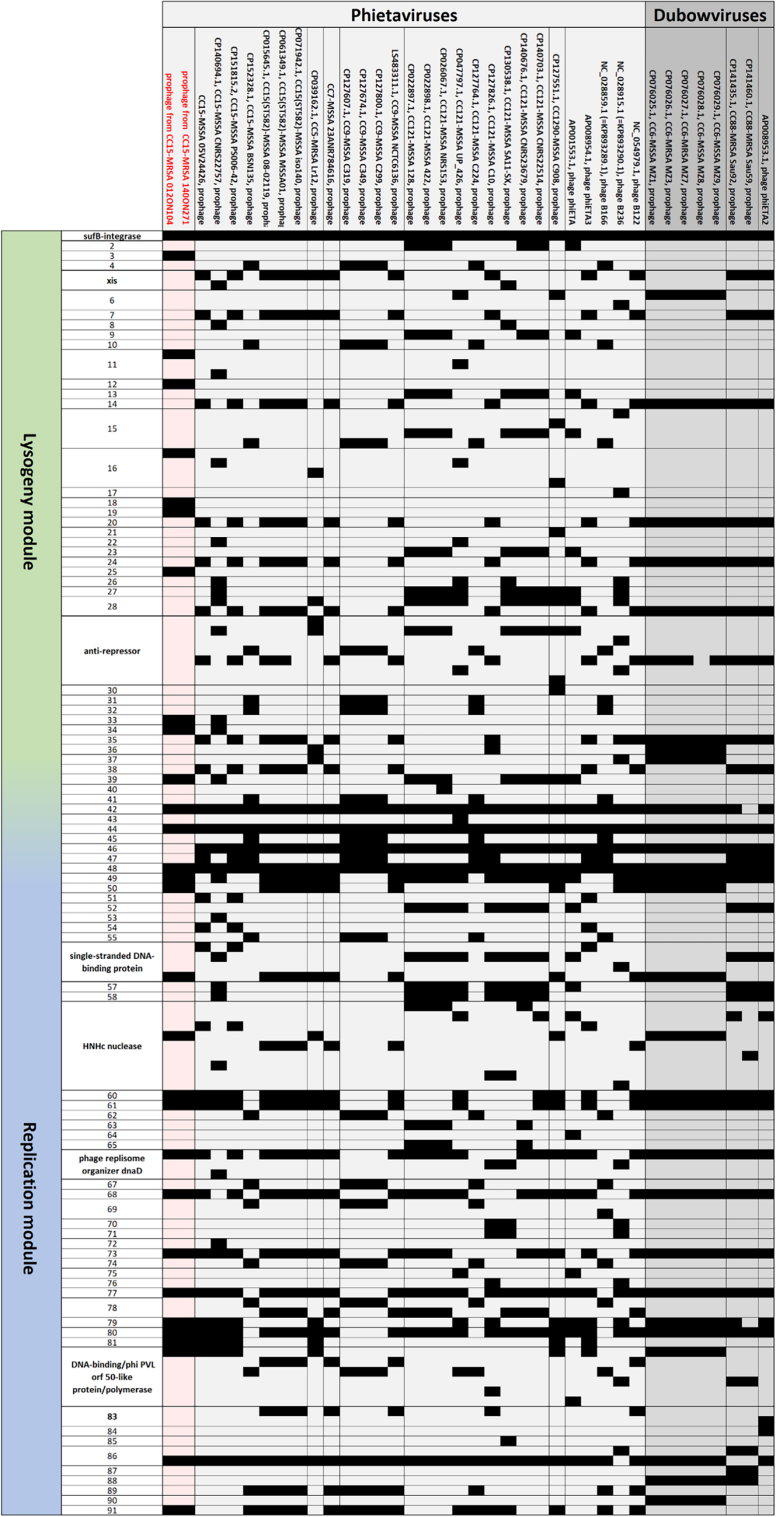

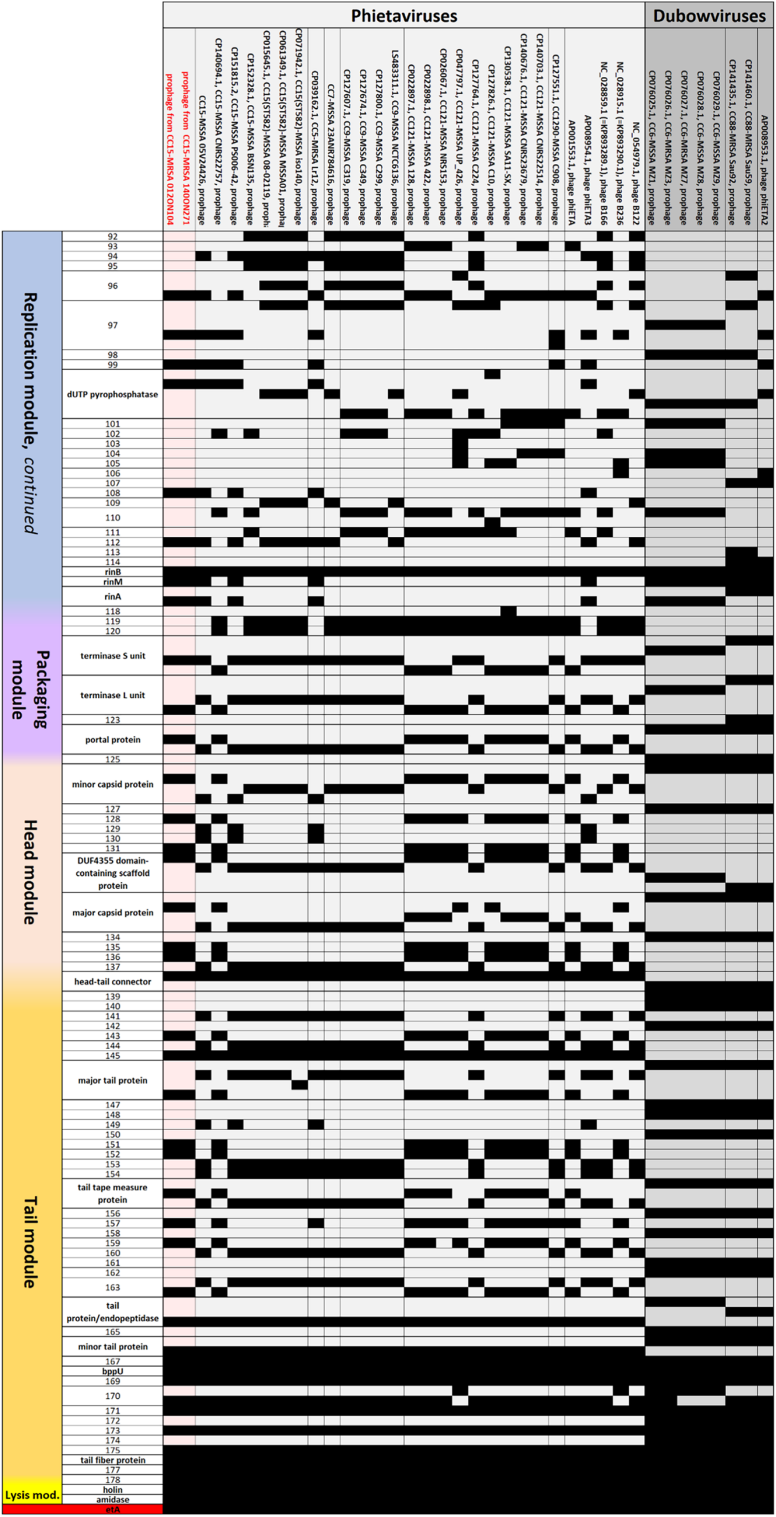
Mapping the gene content of *etA* phages/prophages. All are shown with the integrase gene first, black fields indicate presence of genes/alleles; white ones, absence. The phage sequences as well as full gene designations and locations within the phage sequences are provided in Supplemental Files 5a and b, respectively.

The two Egyptian strains carried each three complete prophage sequences (Supplemental File 4) and one prophage remnant. The most conspicuous prophage was the one carrying the exfoliative toxin A gene *etA*. In both isolates, phage sequences were identical. It was a prophage integrating next to *sufB* with markers known from *Phietavirus* sequences (Supplemental File 4) allowing a presumptive assignment to this genus. A GenBank BLAST search (https://blast.ncbi.nlm.nih.gov/Blast.cgi; as accessed at 2024, Dec. 11th) followed by analysis of the phage/prophage sequences revealed no identical prophages/phages (Supplemental File 5a/b, Fig. 2). The *etA-*prophages from previously published CC15-MSSA sequences were not identical but also can be assigned to the genus *Phietavirus* as a majority of *etA*-phages/prophages (with 28 of the 36 additional sequences analysed belonging to *Phietavirus* while eight were Dubowviruses, Supplemental File 5a/b, Fig. 2).

Furthermore, both Egyptian strains harboured two prophage sequences each with homologies to known *Triavirus* sequences that integrated into the *lip2* = *geh* gene (cgMLST ID: SAUR0317) and into the gene encoding the “putative gene” A5IT17 (cgMLST ID: SAUR1313). Again, these sequences did not differ between the two isolates. The other strains sequenced for comparison lacked obviously the *etA-*carrying prophage although one of them (Kuwait-25912) had a different prophage integrated next to *sufB*. This isolate also carried the same A5IT17-integrating *Triavirus-*like prophage sequence as the two Egyptian isolates.

Both Egyptian, both Kuwaiti and the original Saudi isolate (RUH-2) had an incomplete, truncated prophage integrating into the beta-haemolysin gene *hlb*. It carried the genes for the staphylococcal complement inhibitor and the chemotaxis inhibiting protein (*scn* and *chp*). This phage remnant can be found in most, or possibly even all CC15 sequences (with GenBank CP012970, CP012972, CP120001, CP120009, CP120023, CP149458.1, CP151815, CP152328, and LT963437 being exemplarily analysed).

### Sequencing phage preparations

Nanopore sequencing of phage preparations of both strains, 12ON104 and 140ON271, yielded complete, phage sequences that were identical to the *eta*-positive, *sufB*-integrating *Phietavirus.* In addition, the *lip2*-integrating *Triavirus* sequence was also found in both preparations (Supplemental File 4). These sequences appeared at high coverages (19,800 to 28,800) and, with the exception of the *Triavirus* from 140ON271 (whose sequence also suffered a 1400 nt deletion and a large duplication; Supplemental File 4), they were circular. In addition, both preparations contained phage and genomic fragments, and in the preparation of 12ON104, the plasmid with resistance genes *blaZ, aadD, lnu*(A), *tet*(K) and *cadD/X* was observed. The second, A5IT17-integrating *Triavirus* was not found. Some genes from the *hlb-*integrating prophage remnant were identified on short, low-coverage, non-circular contigs along core genomic markers and thus they were regarded as fragments of chromosomal DNA rather than as induced phages.

### Phage morphology analysis using electron microscopy

In the preparation of 12ON104 and 140ON271, many phage particles with morphological characteristics of *Siphoviridae* were present. Isometric particles – most likely representing *Phietavirus* – and prolate particles – most likely representing *Triavirus* – could be distinguished (Tables 2 and 3). Isometric particles were more numerous than prolate particles. Size and morphology of heads, tails and baseplates of isometric and prolate particles were identical between the preparation of 12ON104 and 140ON271, respectively (Tab. 2 and 3).

**Table 2:** Measurements of the phages exorcised from 12ON104

Type	Head length in nm		Head diameter in nm		Tail diameterin nm		Tail length in nm		Baseplate in nm
	Mean ± SD	Range	Mean ± SD	Range	Mean ± SD	Range	Mean ± SD	Range	Range
Icosahedral phages, *Phietavirus*, *n* = 27	62 ± 3	57–64	60 ± 3	54–64	10 ± 1	9–12	137 ± 16	91–159	25–48 × 19–38
Prolate phages, *Triavirus*, *n* = 10	90 ± 5	83–95	50 ± 6	43–58	–	–	–	–	–

**Table 3:** Measurements of the phages exorcised from 140ON271

Type	Head length in nm		Head diameter in nm		Tail diameter in nm		Tail length in nm		Baseplate in nm
	Mean ± SD	Range	Mean ± SD	Range	Mean ± SD	Range	Mean ± SD	Range	Range
Icosahedral phages, *Phietavirus*, *n* = 24,	62 ± 3	56–64	60 ± 3	54–65	9 ± 1	8–11	139 ± 18	93–160	25–39 × 22–34
Prolate phages, *Triavirus*, *n* = 13	89 ± 15	62–105	48 ± 7	38–60	12, 14	–	308	–	–

**Fig. 3:**
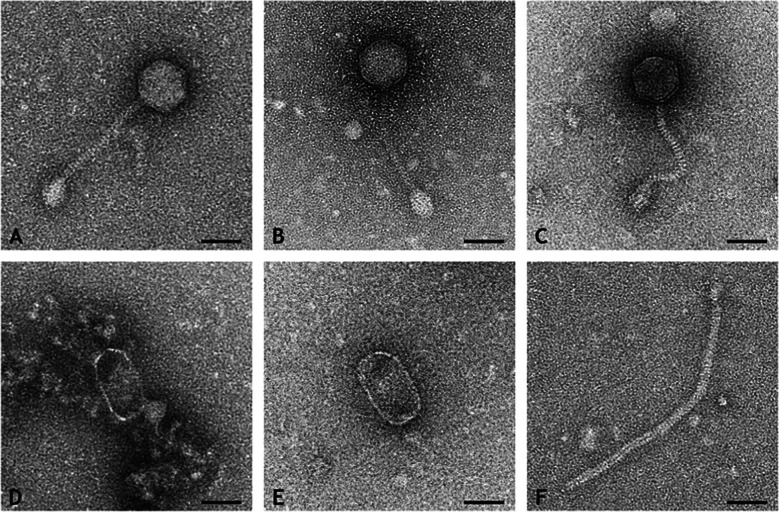
Isometric particles identified as *Phietavirus* and prolate particles compatible with *Triavirus* from phage preparations 12ON104 (**A**, **D**, **F**) and 140ON271 (**B**, **C**, **E**), transmission electron microscopic images of negative contrast preparations with uranyl acetate. Isometric phage particles with identical morphology are present in preparation 12ON104 (**A**) and 140ON271 (**B**). Particles are characterized by icosahedral heads, short thin straight or slightly bent tails of stacked disc appearance and large base plates. Occasionally curved tails are seen (**C**). Prolate particles vary in morphology from thin icosahedral (**D**) to thick oval heads (**E**). Most prolate particles were without tails (**D**, **E**). Isolated long tails most likely belonging to the prolate particles were detected in both preparations (**F**). Bars = 50 nm.

Isometric particles had icosahedral heads, thin, short, straight or curved tails and rather large baseplates (Fig. 3 A, B, C). Prolate particles were quite variable both in length and diameter of their heads ranging between 40 to 50 nm in diameter with icosahedral shape and 50 nm to 60 nm in diameter with oval shape (Fig. 3D, E). Most prolate particles were without tails (Fig. 3D, E). Partial tails were detected in two particles of preparation 140ON271, only. However, isolated tails were present in both preparations which were > 300 nm (Fig. 3F). Since they were markedly longer than those of the isometric particles, these long tails most likely belonged to the prolate particles.

In conclusion, isometric, icosahedral particles of identical morphology were frequently detected in preparations of these isolates from Egypt. The head size was larger than reported in the literature for *Phietavirus*, but otherwise their morphology was consistent to an identification as *Phietavirus*, and given the sequencing results, they might be identified as the *eta*-positive, *sufB*-integrating *Phietavirus.* In addition, prolate particles were detected. Most prolate particles were without tails; thus, they were easy to miss and their number might have been underestimated. Prolate particles were quite variable in size and shape (“thin-icosahedral” and “thick-oval”). This could be consistent with the identification of two different *Triavirus* prophages in the strain´s genomes. However, direct sequencing of the phage preparations showed in both cases only a presence of the *lip2*-integrating *Triavirus* so that this issue remains unresolved.

## Discussion

The study demonstrates a novel livestock-associated MRSA strain from Egypt based on two genome sequences that were essentially identical. It is closely related to a CC15-MRSA-[V + *fus*] strain that emerged some years ago on the Arabian Peninsula but it has now acquired a prophage carrying the exfoliative toxin gene *etA*. In all other relevant features, it is identical to the Middle Eastern strain with notable similarities including the SCC*mec* element, the recombinant *hsdM/S-ssl* locus as well as the gene content of plasmids.

These observations suggest the following evolutionary history of the CC15-MRSA. The recombination affecting the *hsdM/S-ssl* genes was observed in all Saudi, Kuwaiti and Egyptian CC15-MRSA isolates, suggesting a shared ancestor. A GenBank BLAST search (https://blast.ncbi.nlm.nih.gov/Blast.cgi; as accessed at 2025, Jan 17th) revealed two additional genomes sharing this feature, BSN90, GenBank CP149458.1 and BSN135, CP152328.1. Both were CC15-MSSA without SCC-associated genes originating from blood cultures from Detroit, MI, USA. As previously suggested [10], the recombination of the *hsdM/S-ssl* genes might have eventually enabled or facilitated the uptake of foreign mobile genetic elements by CC15 strains leading to emergence of CC15-MRSA after CC15 failed to evolve MRSA for decades. The abovementioned observation far away of the Arabian Gulf region of CC15-MSSA carrying the chimeric *hsdM/S-ssl* genes suggests that the recombination event might indeed have predated the emergence of CC15-MRSA so that the resulting lineage had time to achieve a wider geographic distribution. The evolution to MRSA involved an acquisition of several groups of genes which, as shown above, originated from several different donors. The 5´-terminal part of the SCC element (from *orfX* to *ccrAA*) might have originated from coagulase-negative staphylococci, such as *S. haemolyticus,* which hosts a bewildering variety of “irregular” SCC elements. These genes might have been acquired anytime earlier and elsewhere without being noticed as this recombination did not result in a conspicuous resistance phenotype. The next step, *i.e.,* an acquisition of SCC*mec* and SCC*fus* elements might have happened in the Arabian Gulf region roughly a decade ago, as the evidence from Saudi Arabia suggests. It might also have happened in another region with epidemiological connections to the kingdom, and where the emergence of the strain might have been unnoticed due to a lack of molecular typing and surveillance. This includes Egypt.

SCC*mec* V strains are widespread and common nowadays, and a CC1 strain that harbours a “topologically” similar group of SCC*mec* genes (similar with regard to gene content, order and orientation of genes) has been sequenced from Egypt (GenBank CP113244.1, NZ_JAEOUR000000000.1, NZ_JAEOWK000000000.1 [15, 16];) and it was observed in the Arabian Gulf region [13, 42]. It also had an apparently related *dru* type. SCC-borne *fusC* is increasingly common in the MENA region [11, 13, 16, 43–46]. Similar sets of *fusC*-associated genes have already been sequenced (GenBank MK991791.1, HE980450.1) with one of these sequences even originating from the same time and place as the isolate RUH-2 [40]. The *fusC-*associated gene cluster integrated into the original *S. haemolyticus*-like SCC element, bisecting it and removing several of its genes. As discussed previously [11, 16], the high rate of PVL-positive MRSA in the MENA region might cause a high consumption of fusidic acid ointments as a cure of skin and soft tissue infections. This in turn posed a selective pressure favouring the presence of fusidic acid resistance genes [47]. Strains that carry combined SCC*mec* and SCC*fus* elements thus could have a selective advantage in both, hospital and community settings which might explain their current proliferation in the MENA region.

A final step in the evolution of the Egyptian MRSA strain was an acquisition of prophages. While the *hlb-*integrating prophage remnant is probably present in all CC15 strains, the presence of other prophages varies. The *etA*-carrying prophage was not found in the non-Egyptian isolates of CC15-MRSA-[V + *fus*], neither in those sequenced herein nor in those described elsewhere [10, 13, 14, 43, 44, 48]. The two ST7183/CC15-MRSA-[V + *fus*] sequences in the MLST database (ID 37161and 37162) lack *etA* as previously characterised isolates of CC15-MRSA-[V + *fus*] from a hospital in Alexandria [16]. The *etA* prophage sequence was also not found in a BLAST search of GenBank (https://blast.ncbi.nlm.nih.gov/Blast.cgi; as accessed at 2024, Dec. 11th). Thus, CC15-MRSA-[V + *fus*] must have very recently, during its spread to or in Egypt, acquired an *etA*-prophage from a yet unknown donor. Given the currently published sequences this scenario appears to be more likely than the alternative concept of a pre-existing *etA*-positive CC15-MSSA strain acquiring a SCC*mec*/SCC*fus* composite element.

Phage classification and taxonomy remains a challenge. The morphology-based taxa of staphylococcal phages (*Caudovirales, Siphoviridae*) were abolished with the 2022 taxonomic update of the ICTV bacterial viruses subcommittee, and replaced by a binominal system of nomenclature based on genomic classification [49]. Few data exist that correlate the new species/genera defined by genomic classification with morphologic data. The phage particles which were identified as *Phietavirus* based on sequence data were morphologically—based on the size of the head as well as on the shape of the base plate—more comparable to *Dubowvirus,* which is another member of the family *Azeredovirinae*. More morphological data are required to determine whether all members of a genus defined by genomic classification have similar morphology and whether morphology correlates with sequence.

Further study should investigate distribution and abundance of *etA*-positive CC15-MRSA-[V + *fus*], especially among clinical samples from human infections. It would be interesting to know what are the reasons why *etA-*phages only occur in a few *S. aureus* lineages or what factors limit, or facilitate their transmission. Another interesting issue could be the regulation of the toxin under insufficient antibiotic therapy. It has been noted that a sub-inhibitory concentration of beta-lactams might promote of the expression phage-borne Panton-Valentine leukocidin [50, 51]. It could be important to know, from a clinical/therapeutic point-of-view, whether a similar effect might occur in *etA-*MRSA. Finally, the resistance of the Egyptian strain towards multiple classes of antibiotic compounds, and an emergence of *etA-*positive MRSA in general, should make it necessary to further investigate an adjunct therapeutic use of pooled intravenous immunoglobulins (IVIG) or fresh frozen plasma (FFP) [17]. This approach appears plausible and logical given that antibodies against exfoliative toxins might be present in pooled donor´s blood [17]. While efficacy has been shown at least in paediatric cases [17, 52] there was also a study indicating problems such as longer hospital stays [53].

In conclusion, we describe a novel variant of a CC15 livestock-associated MRSA strain from Egypt. Because of the presence of *etA*, it might be of increased virulence to humans, especially to new-borns who might also be exposed to contaminated milk. Owing to the presence of multiple resistance genes besides *mecA*, therapeutic options are limited. Hence, we urgently recommend surveillance of SSSS/Ritter´s disease in Egypt or in people with relevant travel history.

## Supplementary information

Below is the link to the electronic supplementary material.

ESM 1 (ZIP 680 KB)

ESM 2 (ZIP 982 KB)

ESM 3 (ZIP 1.12 MB)

ESM 4 (FASTA 1.80 MB) 

ESM 5 (ZIP 1.21 MB)

## Data Availability

All relevant information is present in the manuscript and in the Supplemental Files. The GenBank accession numbers of the sequences described are CP191234-CP191243.
