# Supplementary figures and images for: Emergence of livestock-associated MRSA in the Egyptian Nile Delta that carry the exfoliative toxin gene etA: a case for enhanced surveillance
Source: Eur J Clin Microbiol Infect Dis. 2025 Jul 5;44(10):2383–400. doi: 10.1007/s10096-025-05163-z (PMC12484256; doi:10.1007/s10096-025-05163-z)

**Supplemental File 1b:** Alignment of *crrAA* (cassette chromosome recombinase homologue) genes.

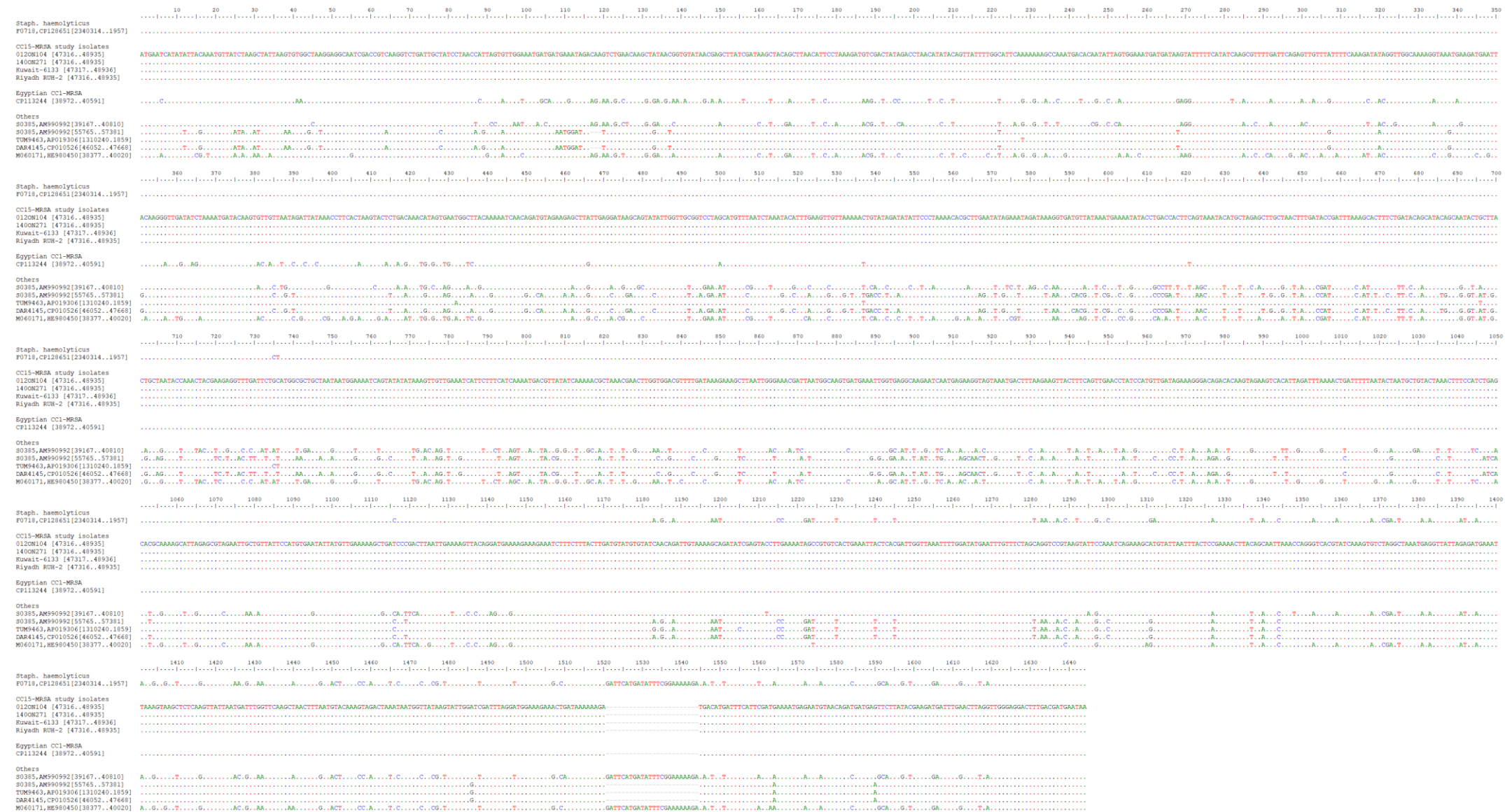

Supplement: Supplementary file 1 — (ZIP 680 KB) [file 10096_2025_5163_MOESM1_ESM.zip › Supplemental File 1b_Alignment ccrAA.pdf]
