# Supplementary material for: Emergence of livestock-associated MRSA in the Egyptian Nile Delta that carry the exfoliative toxin gene etA: a case for enhanced surveillance
Source: Eur J Clin Microbiol Infect Dis. 2025 Jul 5;44(10):2383–400. doi: 10.1007/s10096-025-05163-z (PMC12484256; doi:10.1007/s10096-025-05163-z)

Supplemental File 3b: Alignment of *hsdS* (type I restriction-modification system DNA methylase) genes from *ssl* (νSaα) and *spl* (νSaβ) loci in CC15 genomes.

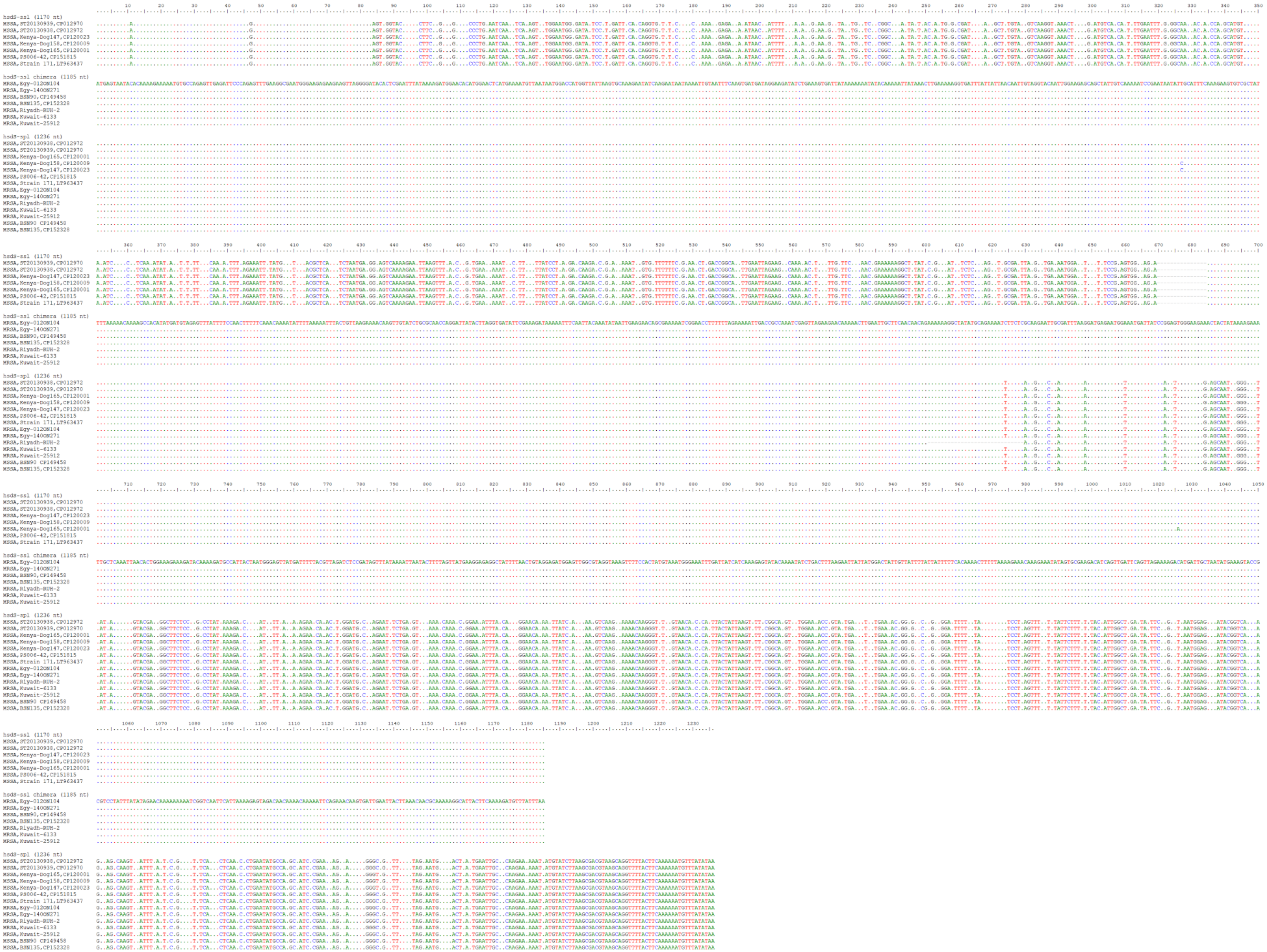

Supplement: Supplementary file 3 — (ZIP 1.12 MB) [file 10096_2025_5163_MOESM3_ESM.zip › Supplemental File 3b_Alignment hsdS in CC15.pdf]
